# Weight Loss Interventions for Breast Cancer Survivors: Impact of Dietary Pattern

**DOI:** 10.1371/journal.pone.0127366

**Published:** 2015-05-26

**Authors:** Henry J. Thompson, Scot M. Sedlacek, Mary C. Playdon, Pamela Wolfe, John N. McGinley, Devchand Paul, Susan G. Lakoski

**Affiliations:** 1 Cancer Prevention Laboratory, Colorado State University, Fort Collins, Colorado, United States of America; 2 Rocky Mountain Cancer Centers, Denver, Colorado, United States of America; 3 Colorado Biostatistics Consortium, University of Colorado Denver, Colorado, United States of America; 4 Department of Internal Medicine, University of Vermont, Burlington, Vermont, United States of America; Mayo Clinic, UNITED STATES

## Abstract

Body weight management is not emphasized in clinical practice guidelines for breast cancer survivors, reflecting the lack of evidence that weight loss improves prognosis. Even if this situation changes, the optimal design for weight loss interventions is unclear. We conducted a 6-month non-randomized, controlled weight loss intervention in 249 post-menopausal breast cancer survivors. This paper reports effects on two secondary endpoints, change in body weight and composition. Participants were predominantly non-Hispanic whites (89%) with a mean age of 54.9 ± 9.2 years, a mean BMI of 29.0 ± 2.6 kg/m: ^2^ and an average of 43 ± 5% body fat. Two dietary interventions, low fat or low carbohydrate, were investigated and consisted of a 42 day cycle of menus and recipes. Weight loss counseling and anthropometric assessment were provided at monthly clinic visits. One hundred ninety-two women completed the trial (77% retention). In comparison to the nonintervention control, both intervention arms achieved significant decreases in body weight (12.5%), body fat (27.5%), waist circumference (9.5%), and hip circumference (7.8%) (all *p* < 0.001) with minimal effects on lean mass (1.3% decrease). Median time to 5 and 10% weight loss was 2 (95% confidence interval = 1 to 3) and 4 (95% confidence interval = 3 to 5) months, respectively, and 23% of participants experienced ≥ 15% weight loss. Loss of body weight and fat mass was rapid and substantial irrespective of dietary approach when a structured program was provided with monthly anthropometric assessment and weight loss counseling.

**Trial Registration:**

ClinicalTrials.gov NCT01315483

## Introduction

Overweight and obesity, which are associated with excessive and abnormal accumulation of body fat, have been reported to worsen prognosis for long term survival following treatment for breast cancer [1]. Available evidence indicates that poorer prognosis is observed when incident cancer occurs in either pre- or post-menopausal overweight or obese women compared to normal weight. The prognostic disadvantage is accounted for by a higher risk of recurrence with subsequent metastatic progression and by the occurrence of cardiovascular disease and type-2 diabetes [2], common co-morbidities of breast cancer survivors. Because the evidence that weight loss improves prognosis is currently considered inadequate [2,3], concern has been expressed in the medical community that giving survivors the task of losing weight represents an unwarranted burden [4]. Thus, body weight management is not emphasized in recently updated clinical practice guidelines despite the fact that the American Society of Clinical Oncology advocates education, awareness, practitioner support, and policy-level change for addressing obesity in the context of cancer prevention and survival [5,6].

The cornerstone of therapeutic interventions to treat or prevent obesity-associated diseases is weight loss via lifestyle modifications involving energy intake and expenditure [7,8]. A number of randomized control trials (RCTs) have been conducted to evaluate various approaches to weight loss and specific dietary patterns have been assessed [9–11]. However, the number of intervention studies of weight loss in breast cancer survivors is still small [12], and as noted in [13], limited sample sizes and short duration of follow-up limit the ability to draw conclusions regarding the most efficacious weight-loss intervention after a breast cancer diagnosis. Moreover, there is little evidence that weight loss programs have been specifically designed for breast cancer survivors, most of whom are considered post-menopausal following completion of treatment [13]. This provided the rationale for focusing on post-menopausal breast cancer survivors in this study and for designing menu plans based on the feedback of breast cancer survivors who participated in a pilot study (Unpublished, HJT).

Many dietary approaches to weight loss have been evaluated in various populations [9,14]. Those that have received the most attention are dietary patterns that are either low carbohydrate or low fat when the macronutrient composition of the diet is expressed as a percent of dietary energy. While interest in macronutrient composition of the diet during weight loss has centered on whether greater weight loss and reduction in percent body fat occurs with diets low in fat versus carbohydrate, the results of a number of RCTs have concluded that these dietary patterns have equivalent effects [9,15]. However, relative to cancer prognosis, emerging but controversial evidence indicates that high glycemic load dietary patterns may increase breast cancer risk [16–22], thus making the comparison of dietary pattern important to investigate in the context of cancer survivorship.

The purpose of this paper is to report on magnitude of weight loss and differences in body composition, both designated secondary endpoints [23], which occurred in breast cancer survivors who followed a 6-month, low fat or low carbohydrate weight loss dietary plan. This study is referred to as CHOICE. The primary endpoint of CHOICE was to determine how weight loss and dietary pattern affect prognostic biomarkers for long-term survival and those findings will be reported separately. Unlike other studies of this type, a 6-week meal plan that was menu- and recipe-defined was provided to each participant. Participants chose from these menus interchangeable, macronutrient-defined (as % energy) and calorie-controlled meals over the 6-month duration of the study. The expectation was that this approach would increase adherence to specific dietary patterns assessed via daily food logs. Moreover, unlike other weight loss studies, anthropometric data were collected monthly, permitting regular adjustment of energy intake and expenditure goals. Monthly clinical visits also reinforced participant accountability for achieving a targeted weight loss objective. A non-randomized design was adopted given concerns that the use of an RCT in dietary interventions may bias potential differences between dietary groups toward the null [24,25]. We judged this particularly important because randomization by dietary pattern may conflict with strong personal dietary preferences, an issue that could be exacerbated in individuals who have undergone cancer therapy.

## Materials and Methods

### Study Design and Participants

The protocol for this trial and supporting CONSORT checklist are available as supporting information; see S1 CONSORT Checklist and S1 Protocol. This study, referred to as CHOICE, was a 6-month non-randomized controlled trial that compared two weight loss interventions, low carbohydrate or low fat, to a nonintervention control. The clinical protocol for CHOICE was described in [23] and the effects of dietary pattern and weight loss on plasma biomarkers of lipid metabolism, which were measured monthly as part of safety monitoring, have also been reported [26]. Potentially eligible women were referred to the research team by their attending physician during a normally scheduled clinical visit and those women who met eligibility criteria were offered participation in the study. Women who enrolled were followed for 6 months in order to create the opportunity for them to achieve a BMI within the normal range and recognizing that weight loss compliance usually decreases for longer periods of time. Anthropometric data (body weight, waist and hip circumference, body mass index, and body composition) were measured monthly. Accrual occurred from 2008 to 2012.

The details of the CHOICE research protocol including eligibility criteria have been published [23]. Briefly, to be eligible, participants were referred by their clinical oncologist, had a pathology report confirming the resected stage of breast cancer and documentation of the type of systemic adjuvant therapy, and had have a BMI in the overweight or obese class I range (BMI 25–34.9 kg/m^2^). In addition, participants: did not anticipate surgery over the study duration period; did not follow a special diet excluding foods or food groups; had not lost 4 or more pounds of body weight over the month preceding study initiation; did not take pharmaceuticals or supplements for weight management; were not being treated for diabetes or blood glucose control; had no history of eating disorders; did not have digestive issues that might interfere with dietary intake, such as irritable bowel syndrome, Crohn’s, or diverticulitis; never had surgery involving constriction or removal of any portion of the gastrointestinal tract; did not have implanted electronic devices such as a pacemaker; and did not use tobacco products. Participants also had to be willing to follow a dietary plan prescribed for the duration of the study; and adhere to American Cancer Society alcohol guidelines (≤1 standard drink per day). Participants were asked to attend 10 one-on-one clinic visits and 5 group visits over 27 weeks and provide 7 fasting blood samples and 3-day pooled urine samples. Enrollment was initiated in 2008 and completed in 2012. Based on work on the primary endpoint, C-reactive protein, published in 2009 [27], and as necessitated by repeated budgetary reductions by the funding agency throughout the clinical trial, statistical power was recomputed for a different end point and the sample size for each group was reduced. This accounts for the lower levels of enrollment relative to those proposed in [23].

### Ethics Statement

The clinical protocol was approved by the Colorado State University Institutional Review Board for the Protection of Human Subjects. Written consent was obtained before enrolling participants.

### Non Intervention Control

Eligible women not interested in participating in the weight loss intervention arms were given the opportunity to enroll in the non-intervention control group. This group was given information about the importance of avoiding post treatment weight gain and the health benefits of having a body mass index in the normal range. Anthropometric data were only collected at baseline and end of study.

### Intervention Designed for Breast Cancer Survivors

Two interventions were designed based on input from participants of a pilot study of weight loss in breast cancer survivors conducted in the same clinical practice in which the intervention was conducted, one year before the intervention study was initiated. That study (Unpublished, HJT) indicated: 1) that a structured diet plan was beneficial to maintaining accountability in losing weight, 2) that the opportunity to evaluate a dietary plan for feasible adaptation to an individual’s taste preferences, which can be altered by cancer treatment, was critical to making a commitment to a program of weight loss, 3) that while a structured weight loss program was critical to reduce cognitive stress, it needed flexibility for life style adaptation and to match culinary abilities, and 4) that many survivors were unable or unwilling to exercise but that increasing activity by walking was highly acceptable. Accordingly, two interventions were developed and were comprised of a structured diet and physical activity program designed to create a weekly negative energy balance equivalent to 3500 kcal, after adjustments for metabolic adaptations that occur during extended periods of weight loss. The intervention groups received the same physical activity protocol promoting 10,000 steps per day and one of two diets that reflect commonly used weight loss approaches that were identified in our pilot study as being of greatest interest to the survivor population. The dietary patterns investigated contained a low percentage of dietary energy as either fat or carbohydrate.

The diet plan for each intervention arm was comprised of a 42-day cycle of menus and recipes. The recipes for each day’s diet plan were entered into ProNutra Diet Analysis software (Version 3.3.0.10, Viocare, Inc., Princeton, NJ) to assure that all breakfast, lunch, or dinner menus had the same percent of dietary energy from protein, fat, and carbohydrate and were therefore interchangeable in this regard. The 42-day cycle menus were designed for five calorie levels in each intervention arm. The meal plans included interchangeable meal options (home-prepared recipes and meal instructions; eating out and convenience meal options), educational material and a program incorporating weight loss strategies. The intervention was designed as a feeding study but was conducted in free living individuals, where strict dietary structure is presented in a format that also offers enough flexibility to be adopted into daily living. To accommodate the importance that survivors in our pilot study placed on acceptability of a dietary plan, and prior to beginning the intervention, participants followed menus and recipes for three days of each intervention and discussed their concerns with the study dietitians. Assignment to intervention arm was made by the project staff based on this dialogue. Since many participants did not have a specific dietary preference, the need to maintain balance in resected disease stage and type of treatment between intervention arms was considered in assignment to the intervention arm and it was possible to maintain balance in study arm assignment throughout the 2008–2012 time span over which the intervention was conducted. Adherence was assessed by the study registered dietitians by monthly level of weight loss as well as daily food record.

### Statistical Methods

Differences in cohort characteristics at baseline across intervention arms and between completers and those lost to follow-up were evaluated using the global F test in a one-way analysis of variance for continuous variables; categorical data were evaluated using a chi-square test for equal proportions. Six-month changes in weight and body composition for each diet group vs control were evaluated in an ANOVA model. For comparing 6-month change between diet groups, an ANCOVA model was used; covariates were baseline BMI, baseline resting metabolic rate, 6-month change in steps, and elapsed time since the end of treatment. The shape of the response curve for weight loss over time was estimated using a maximum likelihood method for repeated measures to accommodate the first order autocorrelation between visits for an individual; the model included baseline BMI, baseline resting metabolic rate, elapsed time since the end of treatment, and the time varying covariate steps; orthogonal polynomial contrasts were used to estimate the shape of the response curve over time. Sensitivity analysis for between-diet differences in 6-month change was done to assess the effects of limiting the comparison to those who completed the 6-month intervention (n = 139) by including all subjects who provided baseline data (n = 167) and a) coding the missing 6-month weight change as 0 or b) using a traditional LOCF (last observation carried forward) analysis; in both cases when 6-month change in steps was missing we used the last recorded value to compute change from baseline. Time-to-event data were evaluated by Kaplan-Meier plots and Cox proportional hazards models. SAS version 9.3 (SAS Institute Inc., Cary, NC) was used for all statistical analyses. GraphPad Prism 5.0 (GraphPad Software, Inc., La Jolla, CA) was used to visualize the data.

## Results

### Study Participants

**Fig 1:**
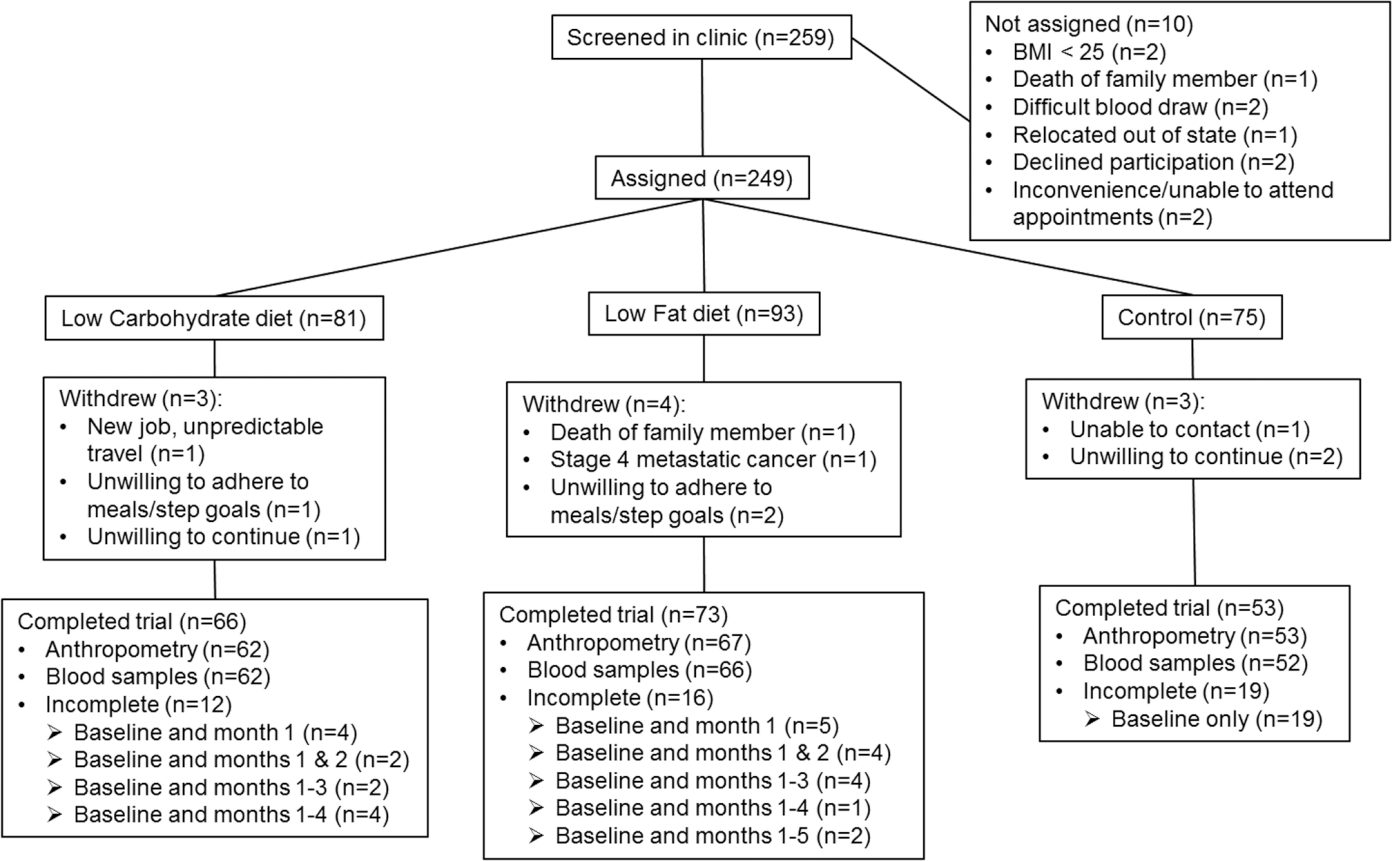
Flow diagram for screening, assignment, and follow-up of the study participants.

A total of 249 participants were assigned to the study (Fig 1). Clinical characteristics and demographic data across groups at baseline are shown in Table 1. Participants were predominately non-Hispanic whites (89%) with a mean age of 54.9 ± 9.2 years, a mean BMI of 29.0 ± 2.6 kg/m^2^ and an average of 43 ± 5% body fat. There were no differences across study arms in clinical or demographic characteristics, including disease stage or treatment regime. During the course of the study, dropout rate was similar in the low carbohydrate (15%) and the low fat (18%) study arms, although it was higher in the non-intervention control (26%); the differences were not statistically significant (*p* = 0.22). Demographics for the 47 cases lost to follow up were not different from those who completed the study, with the exception of time since end of treatment (*p* = 0.01).

**Table 1:** Baseline characteristics.

Variable		Control	Low fat	Low carbohydrate	p-value
		n = 53	n = 73	n = 66	(Global F)
Race	White	50 (94)	70 (96)	72 (94)	0.82
	Black	2 (4)	1 (1)	3 (4)	
	Hispanic	1 (2)	1 (1)	3 (4)	0.46
	Other	1 (2)	2 (3)	1 (2)	
Age (years)		57.7 ± 7.6	54.5 ± 9.2	55.2 ± 8.9	0.11
Height (cm)		164 ± 6	166 ± 6	165 ± 7	0.48
BMI (kg/m^2^)		29.2 ± 2.7	28.2 ± 2.4	29.4 ± 2.5	0.01
Weight (kg)		79.7 ± 9.3	77.6 ± 7.7	79.7 ± 8.6	0.24
Fat Wt (kg)		34.9 ± 7.3	33.0 ± 5.8	35.0 ± 6.0	0.11
Fat Mass (%)		43.5 ± 5.3	42.4 ± 5.2	43.8 ± 4.6	0.24
Lean Wt (kg)		44.8 ± 4.8	44.6 ± 5.2	44.8 ± 5.1	0.97
Lean Mass (%)		56.5 ± 5.3	57.6 ± 5.1	56.3 ± 4.6	0.24
Waist (cm)		95 ± 8	92 ± 7	94 ± 7	0.03
Hip (cm)		111 ± 7	111 ± 6	112 ± 7	0.40
RMR (kcal/d)		1297 ± 132	1284 ± 136	1296 ± 137	0.83
Steps (daily)		6257 ± 3027	7535 ± 2957	7096 ± 2989	0.08

Values are mean ± SD or N (%)

### Outcomes

**Fig 2:**
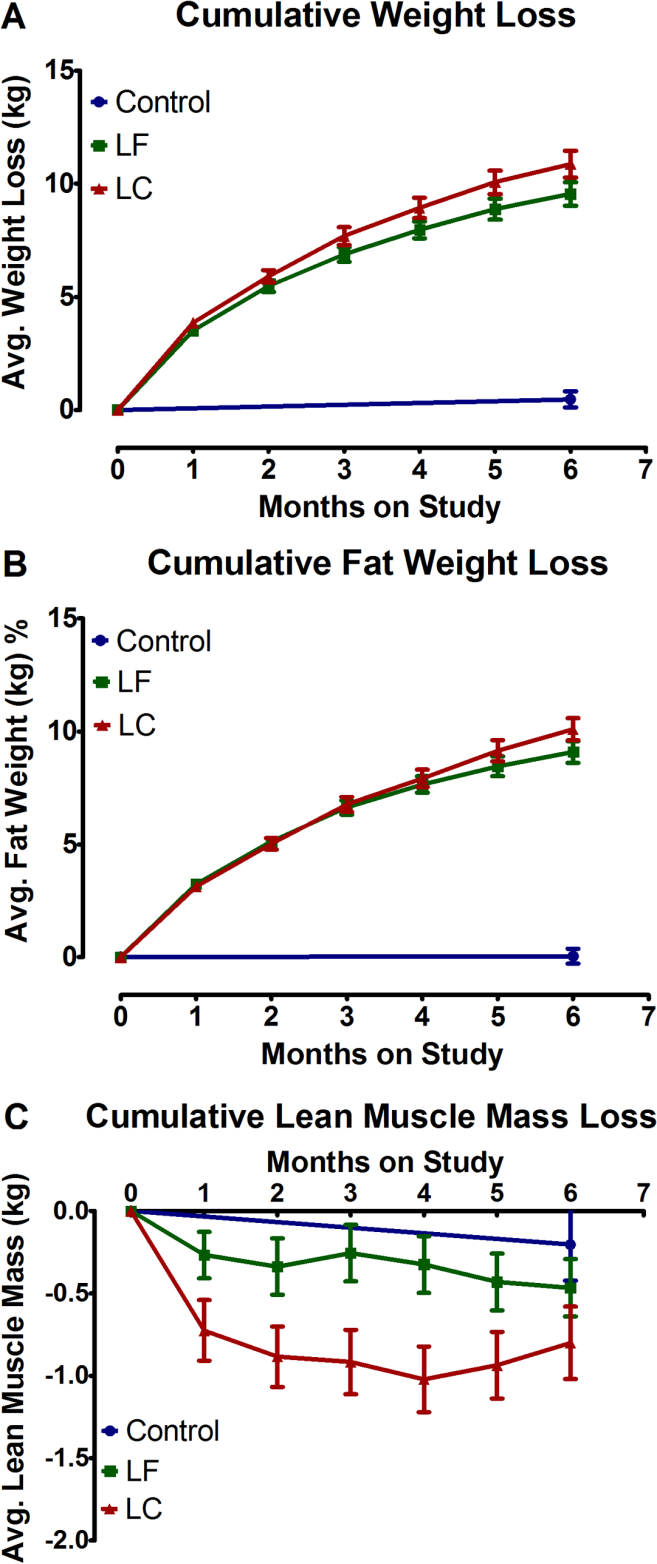
Cumulative Loss of Body Weight, Body Fat, and Lean Body Mass According to Study Group. (A) average cumulative weight loss (kg); (B) average cumulative fat loss (kg); (C) average cumulative loss of lean mass (kg) as a function of time. Values are means ± SEM. LC, low carbohydrate. LF, low fat.

On average the intervention groups lost 9.9 [95% CI = 9.1 to 10.6] kg of body weight, 9.3 [95% CI = 8.6 to 9.9] kg of body fat, 0.6 [95% CI = 0.4 to 0.9] kg of lean weight, 3.7 [95% CI = 3.4 to 3.9] units in body mass index, 8.9 [95% CI = 8.0 to 9.7] cm in waist circumference, and 8.7 [95% CI = 7.9 to 9.5] cm in hip circumference (Table 2). The changes in all parameters, with the exception of lean weight, compared to the changes in the control group were statistically significant (*p* < 0.001) in an unadjusted ANOVA model. The differences between the intervention arms in the primary measures, weight loss, fat weight loss, and lean weight maintenance, gradually diverged over six months. Average cumulative weight loss (kg) (Fig 2A), average cumulative fat loss (kg) (Fig 2B) and average cumulative loss of lean mass (kg) (Fig 2C) are shown as a function of time. Progressive loss of body weight and body fat were best described as a curvilinear (quadratic) response (*p* < 0.01). The cumulative loss of lean mass was greater in the first 2 months, but there was no evidence for a trend over time. An ANCOVA model controlling for baseline BMI, RMR, elapsed time since end of treatment, and the 6-month change in steps, was used to test for between diet differences in6-month percent change (Table 3). None of the measures was statistically significant at the six-month visit. Sensitivity analyses that included all 167 participants in the 2 diet interventions and filled missing outcomes with either the last observation carried forward or 0 where 6-month data were missing also showed no difference between diets.

**Table 2:** Anthropometric measures and body composition.

Variable	Time	Control	Low fat	Low carbohydrate
		(n = 53)	(n = 73)	(n = 66)
Weight (kg)	Baseline	79.7	77.6	79.8
		(77.1 to 82.3)	(75.8 to 79.4)	(77.6 to 81.9)
	6 months	79.4	68.3	69.3
		(76.6 to 82.1)	(66.5 to 70.0)	(67.1 to 71.5)
	Change	-0.4	-9.3	-10.5
		(-1.0 to 0.3)	(-10.3 to -8.3)	(-11.6 to -9.3)
Body Mass Index	Baseline	29.2	28.2	29.4
		(28.5 to 30.0)	(27.6 to 28.8)	(28.7 to 30.0)
	6 months	29.1	24.8	25.5
		(28.3 to 29.9)	(24.2 to 25.3)	(24.8 to 26.1)
	Change	-0.2	-3.4	-3.9
		(-0.4 to 0.1)	(-3.8 to -3.1)	(-4.3 to -3.5)
Fat weight(kg)	Baseline	34.9	33.0	35.0
		(32.9 to 36.9)	(31.6 to 34.3)	(33.5 to 36.5)
	6 months	34.9	24.1	25.3
		(32.6 to 37.1)	(22.8 to 25.5)	(23.7 to 26.9)
	Change	-0.0	-8.9	-9.7
		(-0.7 to 0.6)	(-9.8 to -7.9)	(-10.7 to -8.7)
% Fat Mass	Baseline	43.5	42.4	43.8
		(42.0 to 44.9)	(41.2 to 43.6)	(42.6 to 44.9)
	6 months	43.5	35.1	36.2
		(41.9 to 45.2)	(33.7 to 36.5)	(34.7 to 37.7)
	Change	0.1	-7.3	-7.6
		(-0.5 to 0.6)	(-8.1 to -6.4)	(-8.5 to -6.7)
Lean weight(kg)	Baseline	44.8	44.6	44.8
		(43.5 to 46.1)	(43.4 to 45.8)	(43.5 to 46.0)
	6 months	44.5	44.2	44.0
		(43.1 to 45.8)	(42.9 to 45.4)	(42.7 to 45.2)
	Change	-0.3	-0.4	-0.8
		(-0.7 to 0.0)	(-0.8 to -0.1)	(-1.2 to -0.4)
% Lean Mass	Baseline	56.5	57.6	56.3
		(55.1 to 58.0)	(56.4 to 58.8)	(55.1 to 57.4)
	6 months	56.5	64.9	63.8
		(54.8 to 58.1)	(63.5 to 66.3)	(62.3 to 65.3)
	Change	-0.1	7.3	7.6
		(-0.6, 0.5)	(6.4 to 8.1)	(6.7 to 8.5)
Waist (cm)	Baseline	94.9	91.6	94.2
		(92.6 to 97.2)	(89.9 to 93.3)	(92.5 to 95.9)
	6 months	94.8	83.1	85.0
		(92.4 to 97.2)	(81.3 to 84.8)	(83.1 to 86.8)
	Change	-0.1	-8.5	-9.3
		(-1.6 to 1.4)	(-9.7 to -7.4)	(-10.6 to -7.9)
Hip (cm)	Baseline	110.6	110.7	112.0
		(108.5 to 112.6)	(109.4 to 112.1)	(110.3 to 113.8)
	6 months	111.0	102.2	103.1
		(108.4 to 113.5)	(100.9 to 103.5)	(101.4 to 104.9)
	Change	0.4	-8.6	-8.9
		(-1.1 to 2.0)	(-9.6 to -7.5)	(-10.0 to -7.7)
Waist to Hip Ratio	Baseline	0.86	0.83	0.84
		(0.84 to 0.88)	(0.82 to 0.84)	(0.83 to 0.86)
	6 months	0.86	0.81	0.82
		(0.84 to 0.88)	(0.80 to 0.83)	(0.81 to 0.84)
	Change	-0.00	-0.02	-0.02
		(-0.02 to 0.01)	(-0.02 to -0.01)	(-0.03 to -0.01)
Steps/day	Baseline	6257	7535	7096
		(5358 to 7156)	(6840 to 8230)	(6361 to 7831)
	6 months	n/a	9985	9239
			(9209 to 10760)	(8312 to 10166)
	Change	n/a	2406	2115
			(1622 to 3189)	(1076 to 3154)

Values are means (95% CI). In comparison to the nonintervention control, both intervention arms achieved significant decreases in body weight (12.5%), body fat (27.5%), waist circumference (9.5%), and hip circumference (7.8%) (all *p* < 0.001) with minimal effects on lean mass (1.3% decrease).

**Table 3:** Estimates of between group differences in percent change from baseline for weight loss and body composition at the six month visit.

		Between group difference (LC-LF) Mean ± SEM (*p*-value)	*p*-value			
			Elapsed	Steps	RMR	BMI
% change wt (kg)	CC	0.76 ± 0.91 (0.40)	0.43	0.005	0.47	0.03
	LOCF	0.76 ± 0.92 (0.41)	0.04	0.009	0.49	0.42
	Zero	0.81 ± 1.07 (0.45)	0.02	0.06	0.60	0.73
% change fat wt (kg)	CC	1.96 ± 1.97 (0.32)	0.84	0.001	0.85	0.59
	LOCF	2.23 ± 1.99 (0.26)	0.17	0.003	0.48	0.07
	Zero	1.99 ± 2.31 (0.39)	0.05	0.04	0.56	0.10
% change lean wt (kg)	CC	0.23 ± 0.59 (0.69)	0.60	0.62	0.68	0.18
	AAD	-0.07 ± 0.52 (0.90)	0.35	0.37	0.98	0.08
	Zero	0.27 ± 0.49 (0.57)	0.38	0.57	0.75	0.30

Abbreviations: CC, complete cases (n = 139); LOCF, last observation carried forward (n = 167); Zero, missing 6-month change set to 0 (n = 167); low fat, LF; low carbohydrate, LC; steps, 6-month change in steps; Elapsed, Elapsed time from completion of treatment to initiation of CHOICE; RMR, baseline resting metabolic rate; BMI, baseline body mass index. Inference was done using an ANCOVA model regressing 6-month change on clinically important variables. Note that the sensitivity analysis is conclusive, regardless of the assumptions about missing data the 6-month changes are not different by diet.

### Weight Loss

**Fig 3:**
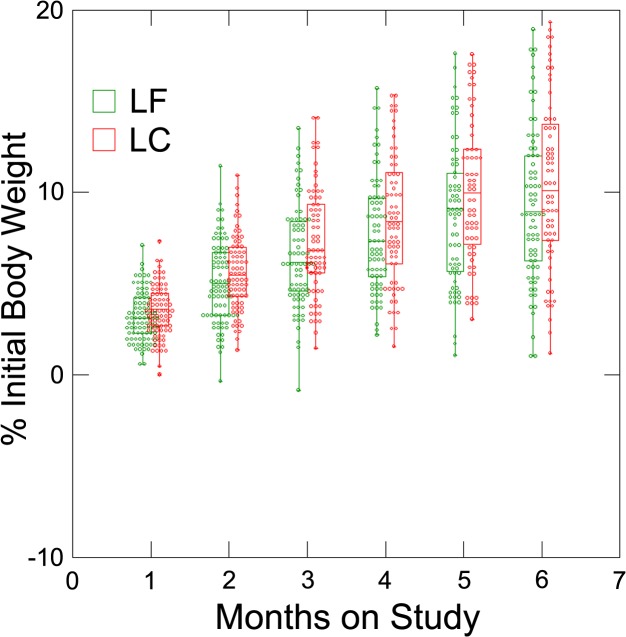
Percent change in Initial Body Weight According to Intervention Arm by Month of Weight Loss. Detailed information on individual success in each intervention arm, box plots of the percent change from initial weight by intervention arm at each of the 6 times points were constructed in a format into which a symmetrical dot density plot was integrated. The box plots show each participant’s data as well as indicating the 25^th^, 50^th^ and 75^th^ percentiles for weight change achieved. These plots show the progressive increments in change in body weight over time by intervention arm and permit a comprehensive view of the magnitude of variation in response.

Box plots were constructed and show the progressive increments in change in percent of initial body weight and the increasing variation in response among individuals over time (Fig 3). Weight loss magnitude at the end of the intervention was 8, 27, 32, 23, and 10%, respectively, for the following categories: <5%, 5.0 to 9.9%, 10.0 to 14.9%, 15.0 to 19.9%, and ≥ 20% weight loss relative to initial weight (Table 4). The differences between intervention arms were not statistically significant.

**Table 4:** Weight Loss Success by Intervention Arm.

	Weight loss, % initial body weight				
Intervention Arm	< 5.0%	5 to 9.9%	10.0 to 14.9%	15.0 to 19.9%	≥ 20.0%
Low fat	6 (8.2%)	23 (31.5%)	24 (32.9%)	15 (20.6%)	5 (6.9%)
Low carbohydrate	5 (7.6%)	15 (22.7%)	20 (30.3%)	17 (25.8%)	9 (13.6%)

Values are n (% of total). The differences between intervention arms were not statistically significant.

**Fig 4:**
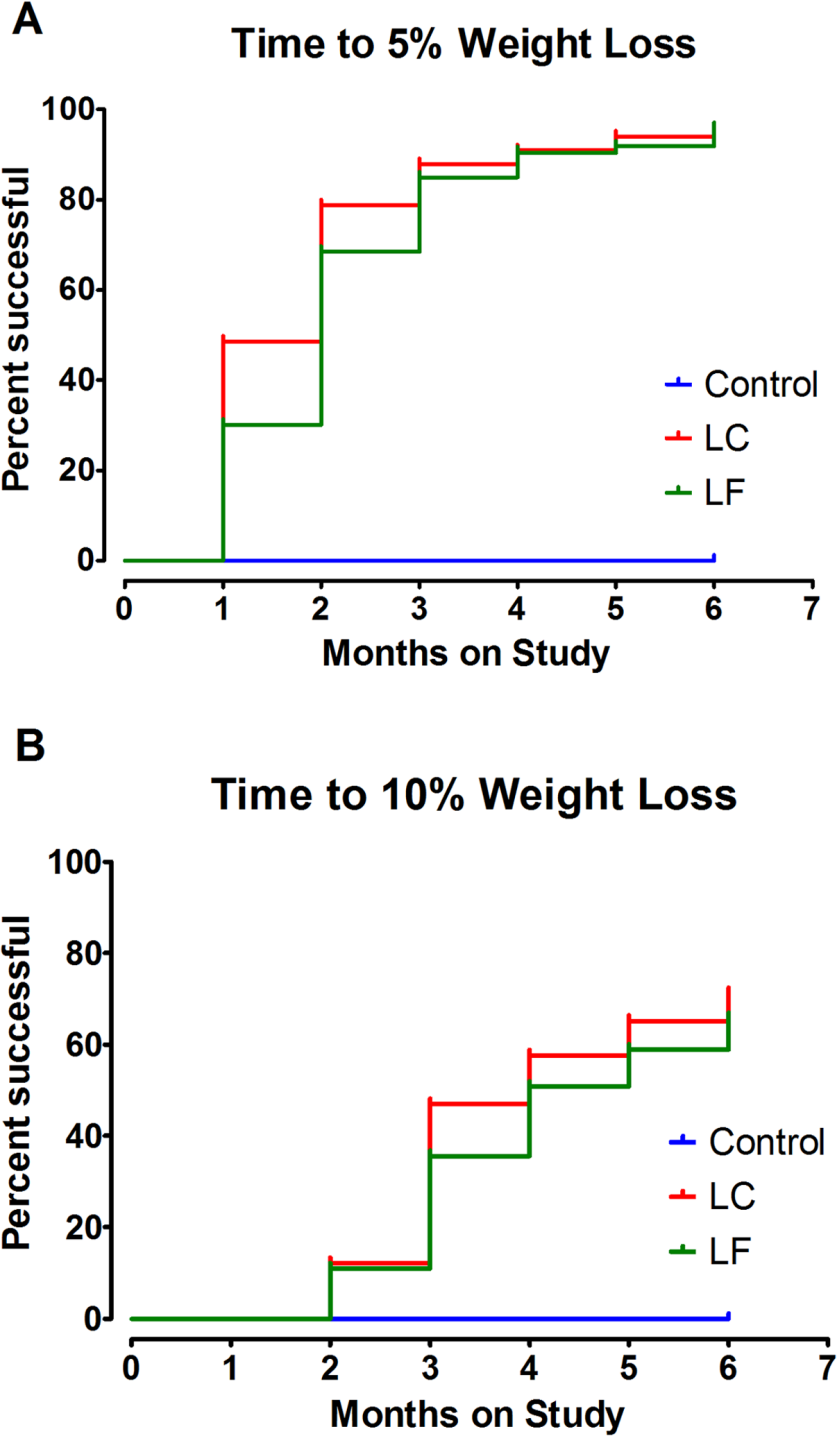
Time-to-Event Analysis for Weight Loss Success According to Intervention Arm. Kaplan-Meier plots were constructed in order to quantify the time frame over which participants in each intervention arm achieved at least a 5%, or 10% reduction in body weight relative to initial body weight. Each plot shows the percent of women in each arm that achieved at least the stated percent weight loss by month. (A) greater than 90% of all women in both intervention arms achieved at least 5% weight loss with a median time to achieving this goal of 2 months (95% CI = 1 to 3 months). Change occurred more rapidly in the low carbohydrate intervention arm but the differences were not statistically significant, tested using a Cox proportional hazard model controlling for BMI, RMR, steps, and elapsed time from end of treatment. (B) median time to loss of ≥ 10% of initial body weight was 4 months (95% CI = 3 to 5 months). LC, low carbohydrate. LF, low fat.

Kaplan-Meier plots (Fig 4) quantify the time frame over which participants in each intervention arm achieved at least a 5 or 10% reduction in body weight relative to initial body weight. Greater than 90% of all women in both intervention arms achieved at least 5% weight loss with a median time to achieving this goal of 2 months (95% CI = 1 to 3 months, Fig 4A). Change occurred more rapidly in the low carbohydrate intervention arm but the differences were not statistically significant using a Cox regression controlling for baseline BMI, baseline RMR, 6 month change in steps and elapsed time since end of treatment; the model was repeated for time to 5% weight loss (p = 0.52), time to 10% weight loss (p = 0.83), time to 15% weight loss (p = 0.39) and time to 20% weight loss (p = 0.40). Median time to loss of ≥ 10% of initial body weight was 4 months (95% CI = 3 to 5 months, Fig 4B)

### Adverse Events

The following adverse events were recorded: treated for a pulmonary embolism (1), treated at an emergency room for stomach pain (1), treated for falls (2), one of which resulted in a hairline hip fracture, and allergic reaction to an antibiotic (1). All adverse events were determined to be not related to the weight loss intervention. Plasma lipid profile [26] and anthropometric data were also monitored monthly and provided no evidence of adverse effects.

## Discussion

The number of breast cancer survivors continues to increase [28]. These women remain at risk for breast cancer recurrence, with survival adversely affected by being overweight or obese [1]. This situation gives urgency to understanding body weight regulation as a potential avenue to improve prognosis [2]. What has been shown in post-menopausal women (not breast cancer survivors) is that limiting caloric intake rather than increasing energy expenditure via physical activity is key to achieving weight loss [29]. In our study, overweight-to-obese postmenopausal breast cancer survivors being routinely followed by a team of medical oncologists working in a private practice setting were remarkably successful in rapid weight loss that was quantified by time-to-event analysis. Weight loss occurred in the absence of adverse events related to the intervention and with little loss of lean mass. The preservation of lean mass is noteworthy and may be due in part to that fact that participants were physically active, although activity levels, measured as daily steps, did not differ between intervention arms. Whether participants were assigned to a low carbohydrate or low fat dietary pattern, average weight loss was 12.5%, which is markedly higher than reported in other studies [13] and only 8% of the study population failed to lose at least 5% of initial body weight, which is a clinically meaningful level of weight loss [30]. This level of success is likely attributed to the fact that the intervention was specifically designed for breast cancer survivors based on their preferences measured during a pilot study. From a clinical practice perspective, the intervention program worked well within the constraints of a large medical oncology practice in the private practice setting (Program details: Table 5).

**Table 5:** Framework for CHOICE as a Transportable Weight Loss Intervention Program.

Intervention Component	Resource	Description
Recruitment	Brochure	Available at front desk reception and medical examination rooms
	Flyers	
Enrollment	Study flow diagram	Description of study components and time frames
	Pictorial	Representation of study rationale
	Welcome letter	
CHOICE Intervention (participant binder)	Introduction	Description of binder contents
	Folder index	
	Meal plan instructions	
	Sample meals	Breakfast, lunch and dinner for low carbohydrate and low fat diets used for assessment of dietary preference
	2-week initiation meal plans	14 days of pre-compiled meals plus full shopping lists
	Interchangeable meal plans	42 days’ worth of interchangeable options for each meal; includes meals requiring recipes plus snack and dessert options
	Cookbook	Recipes and instructions
	Eating out options	Local restaurants and common chain food establishments; 300, 400, 500 and 600 calorie choices
	Frozen meal options	Frozen meals available commercially plus supplementary side dishes and snacks to achieve desired macronutrient content and calorie goals
	Snack Options	100 calorie snack options for addition to lower calorie meal options (e.g. additions to breakfast meal for use as a lunch meal)
	Post Blood Draw Snacks	Snack options appropriate to meal plan and calorie goals following clinic venipuncture
	Quick Reference Guide	Refrigerator magnet reference guide for available meals
	Blank shopping list& meal planning template	Resources to support meal planning and time management
	Exchange list	List of appropriate exchanges for commonly used foods from each food and discretionary food group (meat/ protein, dairy, vegetables, fruits, grains/ cereals, fats, sugars, processed snack items)
	Alcohol-step equivalents	Step equivalents for commonly consumed alcoholic drinks; Daily step goals increased for consumption of any alcoholic beverage
Self Monitoring	One week physical activity and meal record	Completed prior to allocation to intervention or control in order to determine likelihood of compliance
	Meal and activity log	Intervention food record (record meal code plus any deviations from the meal plan); steps/day
	ActiHeart/ Pedometer instructions	Participant instructions for physical activity monitoring
	America On The Move resources	Guide to step equivalents for use in reporting steps/day
	Goal record sheet	Participant diet and physical activity weekly goal record
Educational/ Support Materials	‘Preparing to start your CHOICE diet’	Instructions for preparing to engage in a weight loss program including time and meal management, cooking and food storage preparation, and building social support
	Keeping track handout	Instructions for food, physical activity and weight monitoring, and goal setting
	Weight management handbook	Weight management support resources handbook based on a systematic review of the weight loss literature
	SPRI Xertube and exercise instructions	Guidance on safe home-based resistance training and building exercise capacity
	BMI chart	For use in weight monitoring
	Participant contract	Weight loss contract signed by participant and Registered Dietitian
	Travel nutrition meal plan	Airport meal options, easy travel meals, and travel nutrition tips

A curvilinear response was demonstrated in loss of body fat that directly paralleled the loss of body weight (Table 2; Fig 2B). The changes in body fat composition represent a key finding given the linkages among adiposity, insulin resistance, chronic inflammation, and peripheral aromatization which are metabolic processes implicated not only in breast cancer progression but also in the risk for the common co-morbidities among postmenopausal breast cancer survivors, namely, cardiovascular disease and type-2 diabetes [31]. The changes in waist and hip circumference indicated that fat was being uniformly lost from both peripheral and central fat depots irrespective of the intervention arm to which the participant was assigned.

Despite the success of the current study, there was a broad range in the percent weight loss (Fig 4; Table 4) underscoring the importance of determining whether the magnitude of survival benefit increases or plateaus with increasing weight loss or the achievement of BMI < 25kg/m^2^. While metabolic abnormalities associated with cardiovascular disease and type-2 diabetes, common co-morbidities of breast cancer survivors, are improved with weight loss within the range of 5 to 10% of initial body weight, overall mortality rates and cancer and cardiovascular disease specific death rates are lower in the general population when body mass index is in the normal range (18.5 to 24.9kg/m^2^)[30,32–34]. To our knowledge there are no data to indicate that this relationship does not apply to breast cancer survivors; therefore, we encouraged CHOICE participants to achieve a body mass index of 22 to 23 kg/m^2^ as a target for maximizing survival benefit.

In evaluating the anthropometric data, we recognized that no metrics are generally used to compare the success of weight loss programs, which is particularly important in the private practice setting since the staff resources committed to support weight loss can be considerable. Typical weight loss results (Table 2; Fig 2) fail to provide information about how long it takes for participants to reach weight loss benchmarks, making it difficult to compare various weight loss programs. We tackled this issue recognizing that achieving a loss of initial body weight of 5 or 10% are recognized benchmarks of clinically meaningful weight loss [30]. Time to event analysis was utilized to compare the effects of dietary pattern on achieving these benchmarks. Median time to loss of ≥ 5% of baseline body weight was 2 months (95% CI = 1 to 3 months, Fig 4). Moreover, median time to loss of ≥ 10% of baseline body weight was 4 months (95% CI = 3 to 5 months). Ten percent weight loss is a level of success infrequently observed in weight loss interventions and to our knowledge the speed at which half of the study population reached this standard of success has not been reported in a postmenopausal cohort of cancer survivors [12,13]. Speed or intensity of weight loss is significantly impacts the level of resources that are needed for a successful program.

In evaluating the literature, we also recognized that the magnitude of variation among participants in a weight loss program is difficult to visualize. We found no examples of the characterization of variability among individuals within a cohort during progressive months of a weight loss program. For this purpose, we used box plots into which dot density data were integrated as a graphic visualization tool. The box plot analyses (Fig 3) show that the consistency and magnitude of weight loss declined as the time on study progressed. These data are consistent with the concept that interval-based weight loss might be a more expedient weight loss approach, a concept being evaluated in an ongoing clinical trial in Europe [35].

### Strengths and limitations

This trial had a number of strengths including monthly assessment of body weight and composition during which weight loss counseling occurred, and the use of a structured plan of interchangeable menus and recipes. The use of the print-based meal plan may have been so effective because it was paired with both physical activity and behavior modification (step goals and self-monitoring of weight and diet) and frequent contact with study staff monthly (Summarized in Table 5). Limitations include the fact that the study was neither double blinded nor randomized; thus, the possibility that association between diet intervention and weight loss may have been impacted by unknown or unmeasured confounding factors cannot be ruled out. While the results may not be generalizable to the population as a whole, it can be argued that our findings are more generalizable than standard RCTs in the breast cancer survivor population which is the focus of our work [24,36]. Thus, designing weight loss program based on cancer survivor preferences appears to lead to greater weight loss compared to standard programs [13] and is feasible in this population.

## Conclusions

Clinically meaningful weight loss was achieved in greater than 92% of a population of breast cancer survivors using a program developed and implemented in a private practice setting. Loss of body weight and fat mass was rapid and substantial irrespective of dietary approach when a structured program was provided with monthly anthropometric assessment and weight loss counseling. Given the utility of the CHOICE weight loss program and the limitations of the study design, assessment of the transportability of the intervention approach is required.

## Supporting Information

S1 CONSORT Checklist CONSORT Checklist. (PDF)

S1 Protocol Trial Protocol. (PDF)
