# Supplementary material for: Weight Loss Interventions for Breast Cancer Survivors: Impact of Dietary Pattern
Source: PLoS One. 2015 May 26;10(5):e0127366. doi: 10.1371/journal.pone.0127366 (PMC4443974; doi:10.1371/journal.pone.0127366)
Supplement: S1 Protocol — (PDF) [file pone.0127366.s002.pdf]

Sedlacek et al. BMC Cancer 2011, 11:287
http://www.biomedcentral.com/1471-2407/11/287

BMC Cancer

STUDY PROTOCOL Open Access

Effect of a low fat versus a low carbohydrate weight loss dietary intervention on biomarkers of long term survival in breast cancer patients ('CHOICE'): study protocol

Scot M Sedlacek[1,2], Mary C Playdon[1], Pamela Wolfe[3], John N McGinley[1], Mark R Wisthoff[1], Elizabeth A Daeninck[1], Weiqin Jiang[1], Zongjian Zhu[1] and Henry J Thompson[1*]

Abstract

Background: Weight loss in overweight or obese breast cancer patients is associated with an improved prognosis for long term survival. However, it is not clear whether the macronutrient composition of the chosen weight loss dietary plan imparts further prognostic benefit. A study protocol is presented for a dietary intervention to investigate the effects of weight loss dietary patterns that vary markedly in fat and carbohydrate contents on biomarkers of exposure to metabolic processes that may promote tumorigenesis and that are predictive of long term survival. The study will also determine how much weight must be lost for biomarkers to change in a favorable direction.

Methods/Design: Approximately 370 overweight or obese postmenopausal breast cancer survivors (body mass index: 25.0 to 34.9 kg/m^2) will be accrued and assigned to one of two weight loss intervention programs or a non-intervention control group. The dietary intervention is implemented in a free living population to test the two extremes of popular weight loss dietary patterns: a high carbohydrate, low fat diet versus a low carbohydrate, high fat diet. The effects of these dietary patterns on biomarkers for glucose homeostasis, chronic inflammation, cellular oxidation, and steroid sex hormone metabolism will be measured. Participants will attend 3 screening and dietary education visits, and 7 monthly one-on-one dietary counseling and clinical data measurement visits in addition to 5 group visits in the intervention arms. Participants in the control arm will attend two clinical data measurement visits at baseline and 6 months. The primary outcome is high sensitivity C-reactive protein. Secondary outcomes include interleukin-6, tumor necrosis factor-α, insulin-like growth factor-1 (IGF), IGF binding protein-3, 8-isoprostane-F2-alpha, estrone, estradiol, progesterone, sex hormone binding globulin, adiponectin, and leptin.

Discussion: While clinical data indicate that excess weight for height is associated with poor prognosis for long term survival, little attention is paid to weight control in the clinical management of breast cancer. This study will provide information that can be used to answer important patient questions about the effects of dietary pattern and magnitude of weight loss on long term survival following breast cancer treatment.

Clinical Trial Registration: CA125243

Keywords: biomarkers, dietary patterns, low fat, low carbohydrate, weight loss, breast cancer, long term survival

* Correspondence: henry.thompson@colostate.edu
[1]Cancer Prevention Laboratory, Colorado State University, Fort Collins, CO, USA
Full list of author information is available at the end of the article

BioMed Central

© 2011 Sedlacek et al; licensee BioMed Central Ltd. This is an Open Access article distributed under the terms of the Creative Commons Attribution License (http://creativecommons.org/licenses/by/2.0), which permits unrestricted use, distribution, and reproduction in any medium, provided the original work is properly cited.

Background

Breast cancer is the most common form of cancer in women in the United States [1] and one of the top ten causes of death [2]. Recent estimates show age-adjusted incidence rate is 123.8 per 100,000 women per year [3]. Body fat is now established as being causally related to postmenopausal breast cancer [1,4-8], with overweight or obese women having almost twice the rates of cancer recurrence and up to 1.5 times the risk of death from breast cancer compared to women in the healthy weight range [1,4-7,9-16]. These are alarming findings since the majority of women in the United States are now overweight or obese (i.e. body weight (kg)/height (m^2) > 24.9) [17]. This situation is compounded by the fact that weight gain is common post-diagnosis [18,19].

Studies have shown that losing body weight is protective against breast cancer [20,21], and that weight loss can be achieved through multiple approaches [22]. However, it is unclear whether different diets modeled on popular weight loss programs which vary markedly in macronutrient composition differentially affect long term survival following breast cancer treatment [23-35]. Cross sectional, case control and cohort data is conflicting on fat and carbohydrate intake and breast cancer risk [36-40], with little data available about dietary effects on breast cancer survival [41,42]. Moreover, it is not known whether improvement in biomarkers is progressive with increasing weight loss. This could result in very different clinical guidance related to weight loss in these women.

A number of candidate mechanisms including chronic inflammation [43-46], cellular oxidation [47-57], and insulin resistance [58] may explain the link between energy balance and long term survival following breast cancer treatment. Biomarkers that relate to these mechanisms can be measured in blood and urine to assess potential effects. Although maintaining a healthy body weight is protective against breast cancer and weight loss is feasible in post-menopausal breast cancer survivors [59,60], there are no published studies investigating how fat loss using different dietary macronutrient compositions (i.e. dietary patterns) influences these metabolic and hormonal processes. The CHOICE study seeks to address these questions in an effort to strengthen the evidence base on modifiable lifestyle factors, specifically weight loss, and their effects on long term survival following treatment for breast cancer. Typically, weight loss plateaus after 6-months and most weight is regained after 1 to 5 years [61-64], so in addition to determining whether dietary pattern matters, it will also become critical to identify ways to promote weight loss maintenance so that any protection gained is not transient.

Methods/Design

Study Design

The study, called CHOICE, is a non-randomized, controlled trial in post-menopausal breast cancer survivors investigating whether an energy restricted dietary pattern, i.e. low carbohydrate, high fat or low fat, high carbohydrate, with progressive fat loss during a weight loss program can alter the likelihood of long term survival following treatment for breast cancer as reflected in metabolic and hormonal prognostic biomarkers. A total of 370 women will be accrued and assigned to one of the two dietary intervention arms based on eating preferences or a non-intervention control arm (n = 135 to each intervention arm, n = 100 to the control arm) and followed for 6 months as illustrated in Figure 1. Anthropometric measurements are conducted and biomarkers collected at baseline and 6 months in the control arm, and monthly in the intervention arms in order to model the shape of the metabolic and hormonal response curves.

Study Aims

Primary Aim

The primary aim of the study is to investigate whether dietary pattern (low carbohydrate or low fat) alters the patterns of change observed in circulating factors involved in chronic inflammation, glucose homeostasis, cellular oxidation, and steroid hormone metabolism during progressive loss of body fat. The primary outcome measure is high sensitivity C-reactive protein. All other measurements are considered secondary endpoints.

Secondary Aims

1) To explore whether circulating factors associated with glucose homeostasis, chronic inflammation, and cellular oxidation display similar patterns of change in response to progressive fat loss as circulating hormones associated with sex steroid metabolism.

2) To determine whether either dietary pattern has a differential effect on the magnitude or rate of fat loss and lean tissue changes using a number of devices that permit ongoing and accurate measurements of body composition.

Patient Recruitment (Study Setting and Participants)

CHOICE recruits women who have been treated for breast cancer and attend Rocky Mountain Cancer Centers in Denver, CO, USA. There are approximately 2000 women enrolled in the clinic. Clinic physicians refer breast cancer survivors who meet the eligibility criteria. Participants are also recruited by way of study flyers and brochures posted throughout the clinical facility. Written informed consent is given by subjects when they choose to participate in the program. Following

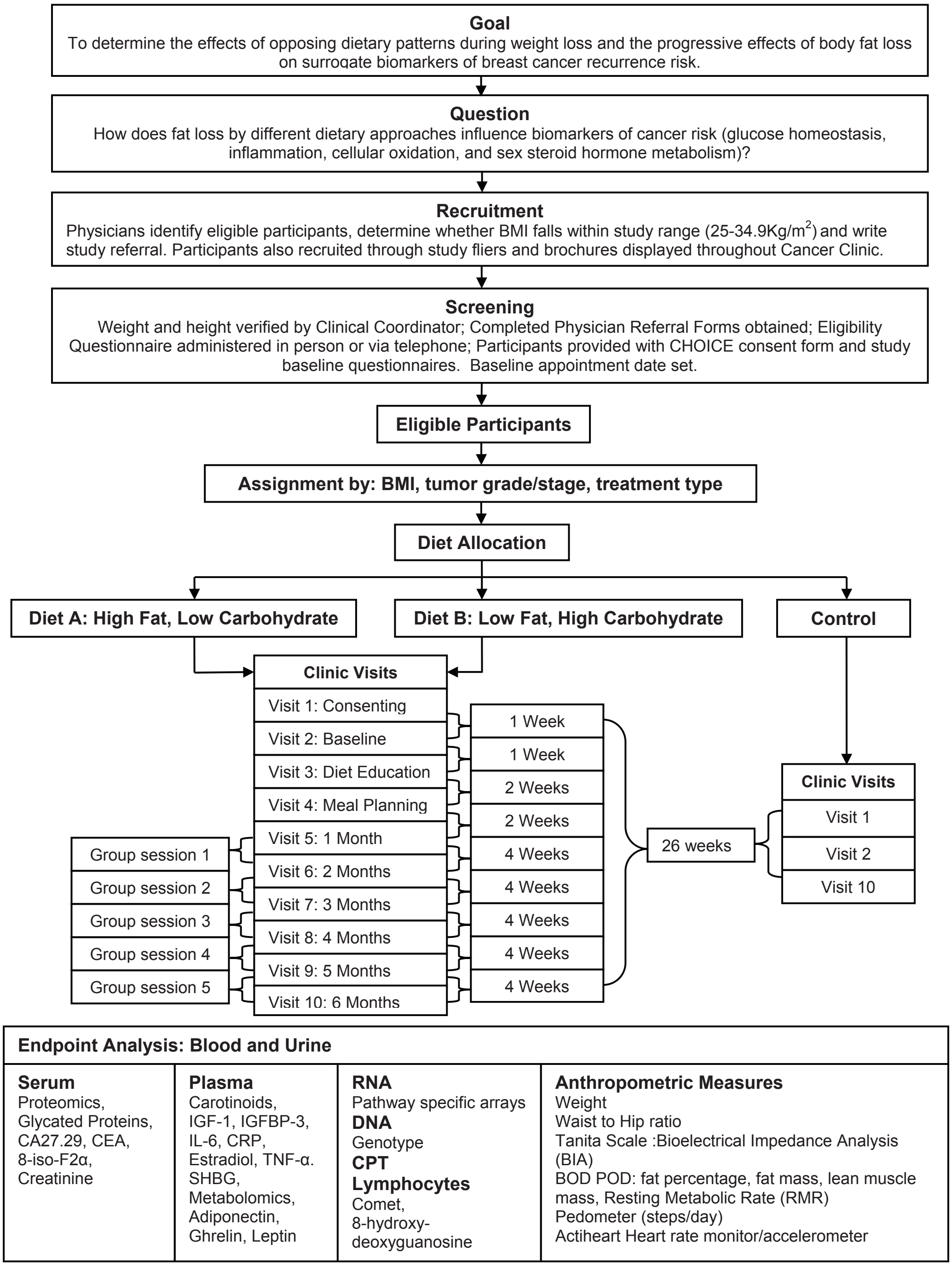

Figure 1 Project Overview

informed consent, participants complete questionnaires that provide demographic, lifestyle and clinical data including age, marital status, ethnicity, level of education, household income, medical history, medicine and supplement use, PAR-Q (Physical Activity Readiness Questionnaire), and emotional well-being. Participants are also interviewed regarding their dieting, weight history and readiness for engaging in a comprehensive weight loss program. This population has a mean age of 60; they are > 98% white and over 50% have post baccalaureate training. While not representative of the general population, this population has proven a very high compliance rate in previous diet-cancer studies conducted by our laboratory [57,65,66].

Eligibility

Participants must be referred by their clinical oncologist, have a pathology report confirming the resected stage of breast cancer and documentation of the type of systemic adjuvant therapy, as well as have a BMI in the overweight or obese class I range (BMI 25-34.9 kg/m^2). Eligible participants: must be at least 4 months post radiation or chemotherapy treatment for breast cancer with no evidence of metastatic disease; must not anticipate having surgery over the study duration period; must not follow a special diet excluding foods or food groups; have not lost 2 or more kg of body weight over the month preceding study initiation; must not be taking pharmaceuticals or supplements for weight management; are not being treated for diabetes or blood glucose control; have no history of eating disorders; do not have digestive issues that may interfere with dietary intake, such as irritable bowel syndrome, Crohn's disease, or diverticulitis; have never had surgery involving constriction or removal of any portion of the gastrointestinal tract; have not been diagnosed with hepatitis B, C, or HIV; do not have implanted electronic devices such as a pacemaker; and do not use tobacco products. Participants must also be willing to follow a dietary plan prescribed for the duration of the study; adhere to American Cancer Society alcohol guidelines (≤ 1 standard drink per day); maintain or increase physical activity as prescribed to achieve negative energy balance required for 0.5-1.0 kg weight loss per week; wear a pedometer and record daily activity; wear an accelerometer/heart rate monitor for 2 weeks during the study (1 week at the beginning and 1 week at the end of the study); wear a body or swim suit and cap for body composition testing; record food intake daily; and attend up to ten one-on-one clinic visits and 5 group visits with seven fasting blood and first-void urine samples in the intervention groups, or three one-on-one clinic visits and two fasting blood and first-void urine samples in the control group over 26 weeks.

Study Groups

Control

Individuals accrued to the non-intervention control group are given the same information currently provided to all breast cancer patients about the importance of avoiding post treatment weight gain, and the health benefits of having a BMI in the normal range. Clinical specimens, questionnaire data, body composition, energy expenditure from activity and anthropometric data are collected at the baseline and 6-month (end of study) visits.

Intervention

Intervention participants follow a fully defined diet-physical activity program designed to create a weekly negative energy balance equivalent to 3500 kcal, after adjustments for metabolic adaptations that occur during extended periods of weight loss. The intervention groups (135 participants in each arm) receive the same physical activity protocol promoting published physical activity guidelines and translated into step recommendations [67,68], but one of two dietary patterns: low fat or low carbohydrate.

Dietary Plans

Dietary patterns are composed of opposing fat and carbohydrate contents but balanced in protein (Table 1). Six-weeks of meal plans were designed for five calorie levels in each diet arm. The meal plans developed included interchangeable meal options with home-prepared recipes and meal instructions. Supporting materials are provided to facilitate adherence including eating out and frozen meal options, food brand options consistent with the plan, meal planning tools and shopping lists. Educational materials were developed based on a systematic review of strategies supporting weight loss maintenance, incorporating program components (e.g. self-monitoring tools) and core competencies reinforcing weight loss behaviors. These strategies are taught to participants through the one-on-one and group sessions in order to promote high levels of dietary adherence.

Participants are instructed to increase their physical activity by increasing steps or step equivalents to contribute to a 500 calorie deficit each day in combination with caloric restriction. Calorie goals are determined based on resting metabolic rate and energy expenditure from activity.

Outcome Measures

C-Reactive Protein

Overweight, obesity and insulin resistance are associated with increases in various cells such as adipocytes, preadipocytes, fibroblasts, and macrophages, which release adipokines and promote inflammation [69-71]. The amount of body fat predicts inflammatory C-reactive protein (CRP) levels among adults [72]. Inflammation decreases apoptosis, increases breast cancer invasiveness and decreases prognosis [46,73]. There is convincing evidence that persistent low grade inflammation as well

Table 1 Mean Proposed Macronutrient Composition by Diet Group

	Low Carbohydrate, High Fat	High Carbohydrate, Low Fat
Carbohydrate (%)	32	64
Fat (%)	48	16
Protein (%)	20	20

as chronic inflammatory diseases are associated with several cancers in humans [44]. Importantly, inflammation measured via CRP is inversely associated with breast cancer survival, even independently of BMI [36,43]. Therefore, CRP is considered the primary outcome.

Secondary Outcomes

Other Markers of Inflammation

Other inflammatory cytokines that mediate the inflammatory response will also be measured, including Interleukin-6 (IL-6) and TNF-α. IL-6 has been correlated with extent of tumor invasion and metastasis [74].

Glucose Homeostasis

As hyperglycemia and insulin resistance are associated with an increased risk for breast cancer [75], factors related to these metabolic processes will be measured including homeostasis model assessment (HOMA). HOMA is a method used to quantify insulin resistance and pancreatic beta cell function and is a calculated index based on fasting levels of insulin and glucose, giving an integrated view of glucose utilization. HOMA has been shown to be associated with increased breast cancer incidence [75], and higher breast cancer mortality in the HEAL study [76]. Fasting insulin alone has also been shown to be predictive of survival in breast cancer patients [77]. In addition, insulin-like growth factor-1 (IGF-1), IGF binding protein-3 (IGFBP-3) and the ratio of IGF-1:IGFBP-3, which provides an estimate of biologically available IGF-1, a more useful indication of overall, longer term control of glucose homeostasis in relation to breast cancer risk, will be measured [78]. Serum IGF-1 is positively associated with breast cancer risk, as well as changes in response to caloric restriction and nutritional alterations [79]. Measuring the effects of two differing dietary patterns on endogenous production of IGF-1 will help to further characterize dietary effects on breast cancer risk.

Cellular Oxidation

The inflammatory response stimulated by obesity and insulin resistance also increases oxidative stress in the body [80]. During oxidative stress, byproducts of nucleic acid metabolism including reactive oxygen species (ROS) promote cancer development by causing genetic mutations and DNA damage [55,56,81]. Secondary measures of cellular oxidation that will be measured include 8-hydroxy-deoxy-guanosine (8-OH-dG) reflecting defects in DNA repair capacity, and markers of whole body lipid peroxidation, 8-isoprostane-F2-alpha (8-iso-$PGF_{2\alpha}$), which have been shown to play a role in breast carcinogenesis [49,82,83]. These byproducts of oxidation are more common in cancerous breast tissue compared to normal breast tissue [49,55,56], and may also be altered with reducing energy intake [51,52] and changes in dietary macronutrient composition [84].

Hormone Metabolism

Overweight postmenopausal women have elevated concentrations of circulating estrogens and lower concentrations of sex hormone binding globulin (SHBG), which promotes cell growth, putting them at more than twice the risk for breast and endometrial cancers, as evidenced in the Healthy Eating and Lifestyles (HEAL) study and the European Prospective Investigation into Cancer and Nutrition (EPIC) study [12,85,86]. Adipose tissue exhibits aromatase enzyme activity, converting androgenic precursors to estradiol and estrone. Estrogens may promote tumorigenesis through direct or indirect induction of free radical-mediated DNA damage, genetic instability, cell mutations and cell proliferation. Agents such as tamoxifen (selective estrogen receptor modulators) or the aromatase inhibitors have been shown to reduce breast cancer incidence and recurrence [87]. Although warranted, there are currently no published randomized, controlled studies of the effect dietary pattern has on estrogens or SHBG and other candidate mechanisms [88-90]. Secondary hormone metabolism outcomes will include estradiol, estrone, progesterone, and SHBG.

Adipokines

Adipose tissue produces the hormones adiponectin and leptin. Low adiponectin, which is an anti-inflammatory and insulin sensitizer, is associated with increased breast cancer mortality in breast cancer survivors [76], with leptin regulating energy balance and metabolism and playing a role in cell proliferation [91]. These adipokines, as well as plasma ghrelin, will be measured to provide biological determinants that may also help explain differences in response to the opposing dietary patterns.

Anthropometry

Weight, height and waist and hip circumferences are measured using a standardized protocol. Participants are in their bathing or body suit for all measurements. Anthropometry is measured monthly in the intervention group and at baseline and 6 months in the control group. Height is measured with a stadiometer. Body circumferences (waist and hip circumferences) are measured using a specially manufactured, tension controlled cloth tape. The participant is instructed to stand erect with arms relaxed at their sides, feet together and abdomen relaxed. Waist circumference is defined as the diameter around the abdomen; it is measured at the midpoint between the top of the hip bone and the

bottom of the rib cage. The measurement is taken at the end of a normal expiration, with the tape pulled tight but not compressing the skin. Hip circumference is measured at the level of the maximum extension of the buttocks.

Body Fat

BOD POD technology is fundamentally the same as underwater (hydrostatic) weighing, but uses volumetric air displacement versus water displacement. All outcome measures including weight are assessed using validated and standardized measuring equipment and techniques. Air displacement plethysmography (BOD POD, Life Measurement, Inc., Concord, CA) has been shown to measure changes in body composition in response to weight change to the same extent as dual x-ray absorptiometry (DEXA), with similar sensitivity [92]. The BOD POD measures the volume of air a person's body displaced while sitting inside a comfortable chamber. By using air versus water, the BOD POD offers a fast, safe, and easy-to-use tool for measuring body composition, without sacrificing accuracy. Since it is based on the same whole-body measurement principle as hydrostatic weighing, the BOD POD first measures the subject's mass and volume. From these measurements, whole-body density is determined, and body fat and lean mass calculated using standardized equations.

Process Measures

Physical Activity

Energy expenditure is measured using accelerometry and heart rate monitoring technology (Actiheart, CamNtech, Inc., Boerne, TX). The Actiheart has been assessed as being valid and reliable in predicting energy expenditure with walking and running [93,94]. Individuals are given a plan to increase physical activity to ultimately meet national guidelines (10,000 steps/d) and facilitate energy balance tailored to their individual lifestyle and physical ability.

Dietary Adherence

There is often a large loss to follow up in lifestyle interventions, especially in longer term studies, which could cause selection bias due to attrition and over-estimation of treatment effects [95]. One method to optimize adherence and retention is to carefully select participants who are more likely to complete the study. The requirements of completing and returning the screening questionnaires, attendance at the individual meetings, the necessity of a clinic visit and the successful completion of the one week food records and other questionnaires are burdensome on potential participants and result in a lower drop-out rate after assignment to a study arm. Additionally, study coordinators stress to potential participants at every screening visit the various obligations required of them in order to take part in this very structured diet study. Potential participants with doubts about participating will be encouraged not to participate in the study or encouraged to participate in the control arm until they are more prepared to make the necessary commitments to the study. The major method for monitoring adherence to the diet is by the use of daily food record with physical activity/step records. Adherence is promoted by 1) ongoing individual contacts in person and by telephone, mail, and email; 2) continuous care/problem solving treatments; 3) skills training to prevent or cope with setbacks; 4) social support and social influence strategies and 5) ease of recording dietary intake by use of meal codes. Additionally, a program of weight management competencies with ongoing assessment and reinforcement has been developed to target the gaps in participants' knowledge and behaviors related to weight loss. During the screening process, participants will be assessed for their readiness to change or maintain change, as well as their general wellbeing or level of depression, which has been shown to be reciprocally associated with obesity and predict weight maintenance success [96].

Monitoring Breast Cancer Recurrence

Women with a history of invasive breast cancer are seen every 3 months for the first 2 years following treatment at the study site clinic. At each visit, a clinical history is updated, a physical examination is performed by the attending oncologist, and serum levels of CA 27.29 and CEA are determined. The frequency of clinical visits decreases to every 6 months for the next three years, and follow up occurs annually thereafter. Following a patient's enrollment into this study, and for the duration over which the project is funded, at each of these clinical visits, the relevant disease recurrence data will be recorded along with height, weight, and body composition. Blood will also be drawn and banked at each visit for subsequent hypothesis testing, although such analyses are beyond the scope of this project.

Data Analysis

All data elements collected will be examined using descriptive statistics (mean, median, SD, range, percentiles, proportion) and graphical summaries (box plots, profile plots by time and diet group). Log transformations will be made before further analysis to stabilize variances as needed.

The primary hypothesis on C-reactive protein will be tested using the following ANCOVA model:

$$\mathbf{Y_4 = a + b_1 Y_1 + b_2 GLYC + e}$$

Where Y_4 is the outcome measure at the end of 6 months, Y_1 is the outcome measure at baseline, and, GLYC is a 2-level indicator for dietary pattern. This method of analysis adjusts for any remaining pre-treatment differences between groups (a precaution against imbalance after diet assignment) and reduces variability in the data being analyzed [97], thus improving the power of the test for the main effect of interest, b_2.

There will be a wealth of information in the repeated measures on each subject; the results for all measures using all available data from all time points will be estimated in a mixed-effects repeated measures model to assess the slopes and between group differences after each month of weight loss [98]. The power of this approach lies in its ability to incorporate all of the available longitudinal data even in the unbalanced case, that is, when some of the observations are missing for one or more individuals. Observations within a person over time are allowed to be correlated while observations across individuals are assumed to be independent. These models will also be used to explore the effects of breast cancer stage, BMI (a time-varying covariate) and age.

Depending on the appearance of change over time (seen in the profile plots of each outcome measure by time and diet) linear or nonlinear mixed models will be used. If the trend appears to be linear, the following model for the response vector $\boldsymbol{y}_i$ for the ith group will be used:

$$\mathbf{y}_i = \mathbf{X}_i\beta + \mathbf{Z}_i\mathbf{b}_i + \mathbf{e}_i \quad (1)$$

where $\mathbf{b}_i \sim N\,(0,\mathrm{D})$ and $\mathbf{e}_i \sim N\,(0,\mathrm{R}_i)$ are independent. X_i is a fixed effects design matrix that includes indicators for diet group (1, 2), assessment time (after each 1.5 kg fat loss, and potential covariates (age) or confounders (disease stage, BMI). $\mathbf{Z}_i$ is a design matrix for the random effects that allows random subject deviations from the population average response. The marginal distribution of $\mathbf{y}_i$ is normal with mean $\mathbf{X}_i\,\beta$ and variance $\mathbf{V}_i = \mathbf{Z}_i\mathbf{D}\mathbf{Z}'_i + \mathbf{R}_i$. Parameter estimation in SAS allows a wide range of specifications for the forms of $\mathbf{D}$ and $\mathbf{R}$, and combines empirical Bayes and restricted maximum likelihood using the EM algorithm.

If the descriptive graphs suggest a nonlinear model is appropriate, we will estimate:

$$\mathbf{y}_i = f\,(\mathbf{X}_i, \beta, \mathbf{Z}_i, \mathbf{b}_i) + \mathbf{e}_i \quad (2)$$

Where $\mathbf{b}_i \sim N\,(\mathbf{0},\mathbf{D})$ and $\mathbf{e}_i \sim N\,(\mathbf{0},\mathbf{R}_i)$. The marginal distribution of $\mathbf{y}_i$ is difficult to find in most cases, but its mean and variance can be approximated by

$$f\,(\mathbf{X}_i, \beta, \mathbf{Z}_i, 0) \text{ and } \mathbf{V}_i = \tilde{\mathbf{Z}}_i\mathbf{D}\tilde{\mathbf{Z}}'_i + \mathbf{R}_i$$

Where $\tilde{\mathbf{Z}}_i$ is the partial derivative of $f\,(\cdot)$ with respect to $\mathbf{b}_i$. Parameter estimation in SAS combines a linearization algorithm, such as Gauss-Newton, and the method of Laird-Ware for linear mixed models. We will explore the use of nonlinear models only if it appears that the response trajectory of Y over time could be fit well by a smooth nonlinear function. Otherwise, simpler piece-wise linear mixed models will be fit using Equation 1 above.

Secondary measures in the 4 families of outcomes will be assessed using the same statistical methods described above for the primary measures. That is, ANCOVA assessment of the measure by diet group at 6 months followed by an exploratory analysis with mixed models using all available data.

Mixed models will also be used to estimate the effects of fat loss on the 4 families of outcomes. Fat loss and weight loss will be modeled with the expectation that differences across diets will be minimal, and success in weight loss better explained by age, initial BMI, and usual level of physical activity.

Missing data are expected to be missing at random, and are unlikely to exceed 5% of all observations. This assumption will be checked during the initial descriptive analysis of the data after database lock, and appropriate sensitivity analyses will be done if there is evidence that the data are MNAR (missing not-at-random) [99]. Given the nature of the patient population and the incentives to return for monthly evaluations, we expect to collect most endpoint measures regardless of compliance. We will accrue 370 subjects with the expectation of completing at least 135 per diet group and 100 in the control group, that is, 10% loss to follow-up. Analysis will be intent-to-treat. All statistical analysis will be done using SAS version 9 (SAS Institute, Cary, NC).

Limitations

Limitations include the fact that the study is neither double blinded nor randomized; results may not be generalizable. The population is free living and as such is subject to issues of compliance and difficulties with quantifying dietary intake and physical activity.

Discussion

A number of reports indicate that the prognosis for long term survival following treatment for breast cancer is better in women whose body weight for height, assessed by body mass index (BMI, body weight (kg)/height (m^2)), is considered to be in the normal range (BMI 18.5 to 24.9) versus women who are overweight (BMI 25.0 to 29.9) or obese BMI ≥ 30.0 [1,4,6,7,9-13,100,101]. Consistent with those reports is the observation that weight gain post diagnosis increases risk for breast cancer recurrence; whereas, weight loss in breast cancer survivors improves the chances of long term survival [20,21]. If one takes the available epidemiological and

clinical data at face value, it prompts the question of why relatively little attention is paid to weight control in the clinical management of breast cancer survivors post treatment.

Overweight and obesity are common problems in the United States, and there is little evidence to indicate that prevalence is less in breast cancer survivors than in the population at large, which is estimated to be > 60% [17]. Obesity has been reported to be the cause for at least 9% breast cancer cases [102]. Thus, given that the majority of breast cancer survivors have excess weight as a risk factor, the population at risk is large. However, a number of challenges are faced by the physician. They include issues such as initiating a conversation about weight loss while recognizing the sensitivity of the subject and time constraints of office visits, which do not allow sufficient time to address the complexity of individual weight management issues, including the knowledge and behavioral gaps related to diet and weight loss. Moreover, there may be hesitation to emphasize weight loss given the recognized 95% long term failure rates of most weight control efforts, making this information a lower priority during the office visit [22,103-110]. Additionally, due to lack of knowledge about the subject matter, basic questions such as, 'How should weight loss be achieved?' and, 'How much weight loss will provide benefit?' cannot be answered with confidence. While many studies have examined differences in effectiveness among various approaches to weight loss [22,103-110], relatively few studies have been conducted in a free living population of breast cancer survivors in the private practice setting. This study will provide information that can be used to answer patient's questions about the effects of dietary pattern and magnitude of weight loss on long term survival following breast cancer treatment.

List of abbreviations

BMI: body mass index; CA 125: cancer antigen 125; CEA: carcinoembryonic antigen; CRP: C-reactive protein; DEXA: dual-energy x-ray absorptiometry; EPIC: European Prospective Investigation into Cancer and Nutrition; HEAL: Health, Eating, Activity and Lifestyle study; HOMA: homeostatic model assessment; IGF: insulin-like growth factor; IGF-BP: insulin-like growth factor binding protein; IL: interleukin; MNAR: missing not-at-random; PAR-Q: physical activity readiness questionnaire; ROS: reactive oxygen species; SHBG: sex hormone binding globulin; TNF-α: tumor necrosis factor alpha; 8-OHdG: 8-hydroxydeoxyguanosine; 8-ISOPGF2α: 8-isoprostaglandin F2 alpha.

Acknowledgements and Funding

United States Public Health Service Grant CA126704 from the National Cancer Institute supported this work.

Author details

[1]Cancer Prevention Laboratory, Colorado State University, Fort Collins, CO, USA. [2]Rocky Mountain Cancer Centers, Denver CO, USA. [3]Colorado Biostatistics Consortium, University of Colorado, Denver, CO, USA.

Authors' contributions

HJT, SMS, PW, JNM, and MRW participated in the design and implementation of the study. MCP, EAD, WJ, ZZ participated in the implementation of the study. All authors participated in the preparation of the manuscript. All authors have read and approved the final manuscript.

Competing interests

The authors declare that they have no competing interests.

Received: 16 June 2011 Accepted: 6 July 2011 Published: 6 July 2011

References

1. Renehan AG, Tyson M, Egger M, Heller RF, Zwahlen M: **Body-mass index and incidence of cancer: a systematic review and meta-analysis of prospective observational studies.** *Lancet* 2008, **371**:569-578.
2. Centers for Disease Control and Prevention (CDC): **Breast Cancer Statistics.** [http://www.cdc.gov/cancer/breast/statistics/index.htm].
3. National Cancer Institute: **SEER Stat Fact Sheet: Breast.**[http://seer.cancer.gov/statfacts/html/breast.html].
4. Daling JR, Malone KE, Doody DR, Johnson LG, Gralow JR, Porter PL: **Relation of body mass index to tumor markers and survival among young women with invasive ductal breast carcinoma.** *Cancer* 2001, **92**:720-729.
5. Huang Z, Willett WC, Colditz GA, Hunter DJ, Manson JE, Rosner B, *et al*: **Waist circumference, waist:hip ratio, and risk of breast cancer in the Nurses' Health Study.** *Am J Epidemiol* 1999, **150**:1316-1324.
6. Petrelli JM, Calle EE, Rodriguez C, Thun MJ: **Body mass index, height, and postmenopausal breast cancer mortality in a prospective cohort of US women.** *Cancer Causes Control* 2002, **13**:325-332.
7. Reeves GK, Pirie K, Beral V, Green J, Spencer E, Bull D: **Cancer incidence and mortality in relation to body mass index in the Million Women Study: cohort study.** *BMJ* 2007, **335**:1134.
8. World Cancer Research Fund/American Institute for Cancer Research.: **Food, Nutrition, Physical Activity, and the Prevention of Cancer: a Global Perspective.** Washington DC, AICR; 2007.
9. Ballard-Barbash R: **Anthropometry and breast cancer. Body size–a moving target.** *Cancer* 1994, **74**:1090-1100.
10. Goodwin PJ, Boyd NF: **Body size and breast cancer prognosis: a critical review of the evidence.** *Breast Cancer Res Treat* 1990, **16**:205-214.
11. La VC, Negri E, Franceschi S, Talamini R, Bruzzi P, Palli D, *et al*: **Body mass index and post-menopausal breast cancer: an age-specific analysis.** *Br J Cancer* 1997, **75**:441-444.
12. McTiernan A: **Obesity and cancer: the risks, science, and potential management strategies.** *Oncology (Williston Park)* 2005, **19**:871-881.
13. McTiernan A, Irwin M, Vongruenigen V: **Weight, physical activity, diet, and prognosis in breast and gynecologic cancers.** *J Clin Oncol* 2010, **28**:4074-4080.
14. Radimer KL, Ballard-Barbash R, Miller JS, Fay MP, Schatzkin A, Troiano R, *et al*: **Weight change and the risk of late-onset breast cancer in the original Framingham cohort.** *Nutr Cancer* 2004, **49**:7-13.
15. Ewertz M, Jensen MB, Gunnarsdottir KA, Hojris I, Jakobsen EH, Nielsen D, *et al*: **Effect of obesity on prognosis after early-stage breast cancer.** *J Clin Oncol* 2011, **29**:25-31.
16. Sinicrope FA, Dannenberg AJ: **Obesity and breast cancer prognosis: weight of the evidence.** *J Clin Oncol* 2011, **29**:4-7.
17. Flegal KM, Carroll MD, Ogden CL, Curtin LR: **Prevalence and trends in obesity among US adults, 1999-2008.** *JAMA* 2010, **303**:235-241.
18. Goodwin PJ, Ennis M, Pritchard KI, McCready D, Koo J, Sidlofsky S, *et al*: **Adjuvant treatment and onset of menopause predict weight gain after breast cancer diagnosis.** *J Clin Oncol* 1999, **17**:120-129.
19. Goodwin PJ: **Weight gain in early-stage breast cancer: where do we go from here?** *J Clin Oncol* 2001, **19**:2367-2369.
20. Harvie M, Howell A, Vierkant RA, Kumar N, Cerhan JR, Kelemen LE, *et al*: **Association of gain and loss of weight before and after menopause with risk of postmenopausal breast cancer in the Iowa women's health study.** *Cancer Epidemiol Biomarkers Prev* 2005, **14**:656-661.
21. Eliassen AH, Colditz GA, Rosner B, Willett WC, Hankinson SE: **Adult weight change and risk of postmenopausal breast cancer.** *JAMA* 2006, **296**:193-201.
22. Alhassan S, Kim S, Bersamin A, King AC, Gardner CD: **Dietary adherence and weight loss success among overweight women: results from the A TO Z weight loss study.** *Int J Obes (Lond)* 2008, **32**:985-991.
23. Alexander DD, Morimoto LM, Mink PJ, Lowe KA: **Summary and meta-analysis of prospective studies of animal fat intake and breast cancer.** *Nutr Res Rev* 2010, 1-11.

24. Blackburn GL, Copeland T, Khaodhiar L, Buckley RB: **Diet and breast cancer.** *J Womens Health (Larchmt)* 2003, **12**:183-192.
25. Gonzalez CA: **The European Prospective Investigation into Cancer and Nutrition (EPIC).** *Public Health Nutr* 2006, **9**:124-126.
26. Michels KB, Mohllajee AP, Roset-Bahmanyar E, Beehler GP, Moysich KB: **Diet and breast cancer: a review of the prospective observational studies.** *Cancer* 2007, **109**:2712-2749.
27. Rohan TE, Negassa A, Caan B, Chlebowski RT, Curb JD, Ginsberg M, *et al*: **Low-fat dietary pattern and risk of benign proliferative breast disease: a randomized, controlled dietary modification trial.** *Cancer Prev Res (Phila Pa)* 2008, **1**:275-284.
28. Larsson SC, Bergkvist L, Wolk A: **Glycemic load, glycemic index and breast cancer risk in a prospective cohort of Swedish women.** *Int J Cancer* 2009, **125**:153-157.
29. Wen W, Shu XO, Li H, Yang G, Ji BT, Cai H, *et al*: **Dietary carbohydrates, fiber, and breast cancer risk in Chinese women.** *Am J Clin Nutr* 2009, **89**:283-289.
30. Lajous M, Boutron-Ruault MC, Fabre A, Clavel-Chapelon F, Romieu I: **Carbohydrate intake, glycemic index, glycemic load, and risk of postmenopausal breast cancer in a prospective study of French women.** *Am J Clin Nutr* 2008, **87**:1384-1391.
31. Cho E, Spiegelman D, Hunter DJ, Chen WY, Colditz GA, Willett WC: **Premenopausal dietary carbohydrate, glycemic index, glycemic load, and fiber in relation to risk of breast cancer.** *Cancer Epidemiol Biomarkers Prev* 2003, **12**:1153-1158.
32. Holmes MD, Liu S, Hankinson SE, Colditz GA, Hunter DJ, Willett WC: **Dietary carbohydrates, fiber, and breast cancer risk.** *Am J Epidemiol* 2004, **159**:732-739.
33. Nielsen TG, Olsen A, Christensen J, Overvad K, Tjonneland A: **Dietary carbohydrate intake is not associated with the breast cancer incidence rate ratio in postmenopausal Danish women.** *J Nutr* 2005, **135**:124-128.
34. Romieu I, Lazcano-Ponce E, Sanchez-Zamorano LM, Willett W, Hernandez-Avila M: **Carbohydrates and the risk of breast cancer among Mexican women.** *Cancer Epidemiol Biomarkers Prev* 2004, **13**:1283-1289.
35. Sieri S, Pala V, Brighenti F, Pellegrini N, Muti P, Micheli A, *et al*: **Dietary glycemic index, glycemic load, and the risk of breast cancer in an Italian prospective cohort study.** *Am J Clin Nutr* 2007, **86**:1160-1166.
36. Pierce JP, Natarajan L, Caan BJ, Parker BA, Greenberg ER, Flatt SW, *et al*: **Influence of a diet very high in vegetables, fruit, and fiber and low in fat on prognosis following treatment for breast cancer: the Women's Healthy Eating and Living (WHEL) randomized trial.** *JAMA* 2007, **298**:289-298.
37. Pierce JP: **Diet and breast cancer prognosis: making sense of the Women's Healthy Eating and Living and Women's Intervention Nutrition Study trials.** *Curr Opin Obstet Gynecol* 2009, **21**:86-91.
38. Pierce JP, Natarajan L, Marshall J, Messer K: **Re: Dietary fat reduction and breast cancer outcome: interim efficacy results from the Women's Intervention Nutrition Study.** *J Natl Cancer Inst* 2007, **99**:900-901.
39. Chlebowski RT: **Lifestyle and breast cancer risk: the way forward?** *J Clin Oncol* 2010, **28**:1445-1447.
40. Chlebowski RT, Blackburn GL: **Diet and breast cancer recurrence.** *JAMA* 2007, **298**:2135-2136.
41. Nichols HB, Trentham-Dietz A, Egan KM, Titus-Ernstoff L, Holmes MD, Bersch AJ, *et al*: **Body mass index before and after breast cancer diagnosis: associations with all-cause, breast cancer, and cardiovascular disease mortality.** *Cancer Epidemiol Biomarkers Prev* 2009, **18**:1403-1409.
42. Trentham-Dietz A, Newcomb PA, Egan KM, Titus-Ernstoff L, Baron JA, Storer BE, *et al*: **Weight change and risk of postmenopausal breast cancer (United States).** *Cancer Causes Control* 2000, **11**:533-542.
43. Pierce BL, Ballard-Barbash R, Bernstein L, Baumgartner RN, Neuhouser ML, Wener MH, *et al*: **Elevated biomarkers of inflammation are associated with reduced survival among breast cancer patients.** *J Clin Oncol* 2009, **27**:3437-3444.
44. Coussens LM, Werb Z: **Inflammation and cancer.** *Nature* 2002, **420**:860-867.
45. Dubois RN: **Leukotriene A4 signaling, inflammation, and cancer.** *J Natl Cancer Inst* 2003, **95**:1028-1029.
46. Schwartsburd PM: **Chronic inflammation as inductor of pro-cancer microenvironment: pathogenesis of dysregulated feedback control.** *Cancer Metastasis Rev* 2003, **22**:95-102.
47. Berwick M, Vineis P: **Markers of DNA repair and susceptibility to cancer in humans: an epidemiologic review.** *J Natl Cancer Inst* 2000, **92**:874-897.
48. Boyd NF, McGuire V: **Evidence of lipid peroxidation in premenopausal women with mammographic dysplasia.** *Cancer Lett* 1990, **50**:31-37.
49. Boyd NF, McGuire V: **The possible role of lipid peroxidation in breast cancer risk.** *Free Radic Biol Med* 1991, **10**:185-190.
50. Breimer LH: **Molecular mechanisms of oxygen radical carcinogenesis and mutagenesis: the role of DNA base damage.** *Mol Carcinog* 1990, **3**:188-197.
51. Cabelof DC, Guo Z, Raffoul JJ, Sobol RW, Wilson SH, Richardson A, *et al*: **Base excision repair deficiency caused by polymerase beta haploinsufficiency: accelerated DNA damage and increased mutational response to carcinogens.** *Cancer Res* 2003, **63**:5799-5807.
52. Cabelof DC, Yanamadala S, Raffoul JJ, Guo Z, Soofi A, Heydari AR: **Caloric restriction promotes genomic stability by induction of base excision repair and reversal of its age-related decline.** *DNA Repair (Amst)* 2003, **2**:295-307.
53. Cerutti PA: **Prooxidant states and tumor promotion.** *Science* 1985, **227**:375-381.
54. Loft S, Vistisen K, Ewertz M, Tjonneland A, Overvad K, Poulsen HE: **Oxidative DNA damage estimated by 8-hydroxydeoxyguanosine excretion in humans: influence of smoking, gender and body mass index.** *Carcinogenesis* 1992, **13**:2241-2247.
55. Malins DC, Haimanot R: **Major alterations in the nucleotide structure of DNA in cancer of the female breast.** *Cancer Res* 1991, **51**:5430-5432.
56. Malins DC, Holmes EH, Polissar NL, Gunselman SJ: **The etiology of breast cancer. Characteristic alteration in hydroxyl radical-induced DNA base lesions during oncogenesis with potential for evaluating incidence risk.** *Cancer* 1993, **71**:3036-3043.
57. Thompson HJ, Heimendinger J, Haegele A, Sedlacek SM, Gillette C, O'Neill C, *et al*: **Effect of increased vegetable and fruit consumption on markers of oxidative cellular damage.** *Carcinogenesis* 1999, **20**:2261-2266.
58. Harvie M, Howell A: **Energy balance adiposity and breast cancer - energy restriction strategies for breast cancer prevention.** *Obes Rev* 2006, **7**:33-47.
59. Chlebowski RT, Aiello E, McTiernan A: **Weight loss in breast cancer patient management.** *J Clin Oncol* 2002, **20**:1128-1143.
60. De WF, Ramlau R, Mulders Y, de VT, van WS: **A feasibility study on weight reduction in obese postmenopausal breast cancer patients.** *Eur J Cancer Prev* 1993, **2**:233-238.
61. Franz MJ, VanWormer JJ, Crain AL, Boucher JL, Histon T, Caplan W, *et al*: **Weight-loss outcomes: a systematic review and meta-analysis of weight-loss clinical trials with a minimum 1-year follow-up.** *J Am Diet Assoc* 2007, **107**:1755-1767.
62. Foster GD, Wadden TA, Kendall PC, Stunkard AJ, Vogt RA: **Psychological effects of weight loss and regain: a prospective evaluation.** *J Consult Clin Psychol* 1996, **64**:752-757.
63. Jeffery RW, Drewnowski A, Epstein LH, Stunkard AJ, Wilson GT, Wing RR, *et al*: **Long-term maintenance of weight loss: current status.** *Health Psychol* 2000, **19**:5-16.
64. Leser MS, Yanovski SZ, Yanovski JA: **A low-fat intake and greater activity level are associated with lower weight regain 3 years after completing a very-low-calorie diet.** *J Am Diet Assoc* 2002, **102**:1252-1256.
65. Thompson HJ, Heimendinger J, Sedlacek S, Haegele A, Diker A, O'Neill C, *et al*: **8-Isoprostane F2alpha excretion is reduced in women by increased vegetable and fruit intake.** *Am J Clin Nutr* 2005, **82**:768-776.
66. Thompson HJ, Heimendinger J, Diker A, O'Neill C, Haegele A, Meinecke B, *et al*: **Dietary botanical diversity affects the reduction of oxidative biomarkers in women due to high vegetable and fruit intake.** *J Nutr* 2006, **136**:2207-2212.
67. Donnelly JE, Blair SN, Jakicic JM, Manore MM, Rankin JW, Smith BK: **American College of Sports Medicine Position Stand. Appropriate physical activity intervention strategies for weight loss and prevention of weight regain for adults.** *Med Sci Sports Exerc* 2009, **41**:459-471.
68. Haskell WL, Lee IM, Pate RR, Powell KE, Blair SN, Franklin BA, *et al*: **Physical activity and public health: updated recommendation for adults from the American College of Sports Medicine and the American Heart Association.** *Med Sci Sports Exerc* 2007, **39**:1423-1434.
69. van Kruijsdijk RC, van der Wall E, Visseren FL: **Obesity and cancer: the role of dysfunctional adipose tissue.** *Cancer Epidemiol Biomarkers Prev* 2009, **18**:2569-2578.
70. Libby P: **Inflammation in atherosclerosis.** *Nature* 2002, **420**:868-874.
71. Yudkin JS, Stehouwer CD, Emeis JJ, Coppack SW: **C-reactive protein in healthy subjects: associations with obesity, insulin resistance, and**

endothelial dysfunction: a potential role for cytokines originating from adipose tissue? *Arterioscler Thromb Vasc Biol* 1999, **19**:972-978.
72. Wener MH, Daum PR, McQuillan GM: **The influence of age, sex, and race on the upper reference limit of serum C-reactive protein concentration.** *J Rheumatol* 2000, **27**:2351-2359.
73. Goldberg JE, Schwertfeger KL: **Proinflammatory cytokines in breast cancer: mechanisms of action and potential targets for therapeutics.** *Curr Drug Targets* 2010, **11**:1133-1146.
74. Ravishankaran P, Karunanithi R: **Clinical significance of preoperative serum interleukin-6 and C-reactive protein level in breast cancer patients.** *World J Surg Oncol* 2011, **9**:18.
75. Sieri S, Muti P, Claudia A, Berrino F, Pala V, Grioni S, *et al*: **Prospective study on the role of glucose metabolism in breast cancer occurrence.** *Int J Cancer* 2011.
76. Duggan C, Irwin ML, Xiao L, Henderson KD, Smith AW, Baumgartner RN, *et al*: **Associations of insulin resistance and adiponectin with mortality in women with breast cancer.** *J Clin Oncol* 2011, **29**:32-39.
77. Goodwin PJ, Ennis M, Pritchard KI, Trudeau ME, Koo J, Madarnas Y, *et al*: **Fasting insulin and outcome in early-stage breast cancer: results of a prospective cohort study.** *J Clin Oncol* 2002, **20**:42-51.
78. Goodwin PJ, Ennis M, Pritchard KI, Trudeau ME, Koo J, Hartwick W, *et al*: **Insulin-like growth factor binding proteins 1 and 3 and breast cancer outcomes.** *Breast Cancer Res Treat* 2002, **74**:65-76.
79. Key TJ: **Glycemic index, hyperinsulinemia, and breast cancer risk.** *Ann Oncol* 2001, **12**:1507-1509.
80. Erlinger TP, Platz EA, Rifai N, Helzlsouer KJ: **C-reactive protein and the risk of incident colorectal cancer.** *JAMA* 2004, **291**:585-590.
81. Thompson HJ: **DNA oxidation products, antioxidant status, and cancer prevention.** *J Nutr* 2004, **134**:3186S-3187S.
82. Hoeijmakers JH: **Genome maintenance mechanisms for preventing cancer.** *Nature* 2001, **411**:366-374.
83. Hoeijmakers JH: **DNA repair mechanisms.** *Maturitas* 2001, **38**:17-22.
84. Thompson HJ, Heimendinger J, Gillette C, Sedlacek SM, Haegele A, O'Neill C, *et al*: **In vivo investigation of changes in biomarkers of oxidative stress induced by plant food rich diets.** *J Agric Food Chem* 2005, **53**:6126-6132.
85. Cauley JA, Gutai JP, Kuller LH, LeDonne D, Powell JG: **The epidemiology of serum sex hormones in postmenopausal women.** *Am J Epidemiol* 1989, **129**:1120-1131.
86. Kaaks R, Rinaldi S, Key TJ, Berrino F, Peeters PH, Biessy C, *et al*: **Postmenopausal serum androgens, oestrogens and breast cancer risk: the European prospective investigation into cancer and nutrition.** *Endocr Relat Cancer* 2005, **12**:1071-1082.
87. Renehan AG, Roberts DL, Dive C: **Obesity and cancer: pathophysiological and biological mechanisms.** *Arch Physiol Biochem* 2008, **114**:71-83.
88. Cardona-Gomez GP, Mendez P, DonCarlos LL, Azcoitia I, Garcia-Segura LM: **Interactions of estrogens and insulin-like growth factor-I in the brain: implications for neuroprotection.** *Brain Res Brain Res Rev* 2001, **37**:320-334.
89. Mejia-Naranjo W, Yakar S, Bernal R, Leroith D, Sanchez-Gomez M: **Regulation of the splenic somatotropic axis by dietary protein and insulin-like growth factor-I in the rat.** *Growth Horm IGF Res* 2003, **13**:254-263.
90. Mendez P, Cardona-Gomez GP, Garcia-Segura LM: **Interactions of insulin-like growth factor-I and estrogen in the brain.** *Adv Exp Med Biol* 2005, **567**:285-303.
91. Jarde T, Perrier S, Vasson MP, Caldefie-Chezet F: **Molecular mechanisms of leptin and adiponectin in breast cancer.** *Eur J Cancer* 2011, **47**:33-43.
92. Weyers AM, Mazzetti SA, Love DM, Gomez AL, Kraemer WJ, Volek JS: **Comparison of methods for assessing body composition changes during weight loss.** *Med Sci Sports Exerc* 2002, **34**:497-502.
93. Brage S, Brage N, Franks PW, Ekelund U, Wareham NJ: **Reliability and validity of the combined heart rate and movement sensor Actiheart.** *Eur J Clin Nutr* 2005, **59**:561-570.
94. Brage S, Brage N, Ekelund U, Luan J, Franks PW, Froberg K, *et al*: **Effect of combined movement and heart rate monitor placement on physical activity estimates during treadmill locomotion and free-living.** *Eur J Appl Physiol* 2006, **96**:517-524.
95. Dansinger ML, Tatsioni A, Wong JB, Chung M, Balk EM: **Meta-analysis: the effect of dietary counseling for weight loss.** *Ann Intern Med* 2007, **147**:41-50.
96. Luppino FS, de Wit LM, Bouvy PF, Stijnen T, Cuijpers P, Penninx BW, *et al*: **Overweight, obesity, and depression: a systematic review and meta-analysis of longitudinal studies.** *Arch Gen Psychiatry* 2010, **67**:220-229.
97. Fleiss JL: *Design and Analysis of Clinical Experiments* New York, NY: John Wiley & Sons; 1986.
98. Verbeke G, Molenberghs G: *Linear Mixed Models in Practice:A SAS-Oriented Approach* New York: Springer-Verlag; 1997.
99. Fairclough D: *Design and Analysis of Quality of Life Studies in Clinical Trials* Boca Raton: Chapman & Hall/CRC; 2002.
100. Huang Z, Willett WC, Colditz GA, Hunter DJ, Manson JE, Rosner B, *et al*: **Waist circumference, waist:hip ratio, and risk of breast cancer in the Nurses' Health Study.** *Am J Epidemiol* 1999, **150**:1316-1324.
101. Radimer KL, Ballard-Barbash R, Miller JS, Fay MP, Schatzkin A, Troiano R, *et al*: **Weight change and the risk of late-onset breast cancer in the original Framingham cohort.** *Nutr Cancer* 2004, **49**:7-13.
102. Wolin KY, Carson K, Colditz GA: **Obesity and cancer.** *Oncologist* 2010, **15**:556-565.
103. **Position of the American Dietetic Association: weight management.** *J Am Diet Assoc* 1997, **97**:71-74.
104. Sacks FM, Bray GA, Carey VJ, Smith SR, Ryan DH, Anton SD, *et al*: **Comparison of weight-loss diets with different compositions of fat, protein, and carbohydrates.** *N Engl J Med* 2009, **360**:859-873.
105. Bravata DM, Sanders L, Huang J, Krumholz HM, Olkin I, Gardner CD, *et al*: **Efficacy and safety of low-carbohydrate diets: a systematic review.** *JAMA* 2003, **289**:1837-1850.
106. Greene LF, Malpede CZ, Henson CS, Hubbert KA, Heimburger DC, Ard JD: **Weight maintenance 2 years after participation in a weight loss program promoting low-energy density foods.** *Obesity (Silver Spring)* 2006, **14**:1795-1801.
107. Brehm BJ, Seeley RJ, Daniels SR, D'Alessio DA: **A randomized trial comparing a very low carbohydrate diet and a calorie-restricted low fat diet on body weight and cardiovascular risk factors in healthy women.** *J Clin Endocrinol Metab* 2003, **88**:1617-1623.
108. Grieb P, Klapcinska B, Smol E, Pilis T, Pilis W, Sadowska-Krepa E, *et al*: **Long-term consumption of a carbohydrate-restricted diet does not induce deleterious metabolic effects.** *Nutr Res* 2008, **28**:825-833.
109. Adam-Perrot A, Clifton P, Brouns F: **Low-carbohydrate diets: nutritional and physiological aspects.** *Obes Rev* 2006, **7**:49-58.
110. Nordmann AJ, Nordmann A, Briel M, Keller U, Yancy WS Jr, Brehm BJ, *et al*: **Effects of low-carbohydrate vs low-fat diets on weight loss and cardiovascular risk factors: a meta-analysis of randomized controlled trials.** *Arch Intern Med* 2006, **166**:285-293.

Pre-publication history

The pre-publication history for this paper can be accessed here:
http://www.biomedcentral.com/1471-2407/11/287/prepub

doi:10.1186/1471-2407-11-287
Cite this article as: Sedlacek *et al.*: Effect of a low fat versus a low carbohydrate weight loss dietary intervention on biomarkers of long term survival in breast cancer patients ('CHOICE'): study protocol. *BMC Cancer* 2011 **11**:287.

Submit your next manuscript to BioMed Central and take full advantage of:

- **Convenient online submission**
- **Thorough peer review**
- **No space constraints or color figure charges**
- **Immediate publication on acceptance**
- **Inclusion in PubMed, CAS, Scopus and Google Scholar**
- **Research which is freely available for redistribution**

Submit your manuscript at
www.biomedcentral.com/submit

BioMed Central
